# Interplay between stress granule-like structures and nuclear RNA export in a heat shock-specific manner

**DOI:** 10.1093/nar/gkag840

**Published:** 2026-08-20

**Authors:** Hosung Jeon, Donghwan Shin, Yeongyo Son, Jiyeun Park, Dong Jae Lee, Jae Sung Shim, Dae-Geun Song, Hokyoung Son

**Affiliations:** Department of Agricultural Biotechnology, Seoul National University, Seoul 08826, Republic of Korea; Department of Agricultural Biotechnology, Seoul National University, Seoul 08826, Republic of Korea; Department of Agricultural Biotechnology, Seoul National University, Seoul 08826, Republic of Korea; Research Institute of Agriculture and Life Sciences, Seoul National University, Seoul 08826, Republic of Korea; Institute for Plant Sciences, University of Cologne, Cologne 50674, Germany; Center for Natural Product Systems Biology, Korea Institute of Science and Technology (KIST) Gangneung Institute of Natural Products, Gangneung 25451, Republic of Korea; School of Biological Sciences and Technology, Chonnam National University, Gwangju 61186, Republic of Korea; Institute of Plant Biomaterials and Biomedical Science, Chonnam National University, Gwangju 61186, Republic of Korea; Center for Natural Product Systems Biology, Korea Institute of Science and Technology (KIST) Gangneung Institute of Natural Products, Gangneung 25451, Republic of Korea; Natural Product Applied Science, KIST School, University of Science and Technology, Gangneung 25451, Republic of Korea; Department of Agricultural Biotechnology, Seoul National University, Seoul 08826, Republic of Korea; Research Institute of Agriculture and Life Sciences, Seoul National University, Seoul 08826, Republic of Korea; Plant Health Center, Seoul National University, Seoul 08826, Republic of Korea; Plant Genomics and Breeding Institute, Seoul National University, Seoul 08826, Republic of Korea

## Abstract

When stress strikes, cells initiate adaptive responses, including perturbation of RNA splicing, modulation of nuclear RNA export, and formation of stress granules (SGs). Despite their central role, the composition and regulation of fungal SGs remain poorly understood. Here, we report the formation of heat shock-induced SG-like structures in *Fusarium graminearum*. Through purification of SG-like structures followed by RNA-sequencing, we performed a transcriptome-wide characterization of their associated transcripts and found that heat shock induces a distinct enrichment of intron-containing transcripts within SG-like structures compared to physiological conditions. Moreover, these SG-like structures co-localize with the nuclear RNA export factor FgYra1 in the nucleus, linking SG-like structures to the RNA export system. Furthermore, transcripts associated with SG-like structures exhibit distinct nuclear retention behaviors during heat shock, and the loss of *FgYRA1* alters nuclear retention in an atypical manner. Together, this study defines the molecular architecture of *F. graminearum* SG-like structures and highlights their functional relevance in coordinating nuclear RNA export during heat shock.

## Graphical Abstract

**Graphical Abstract:**
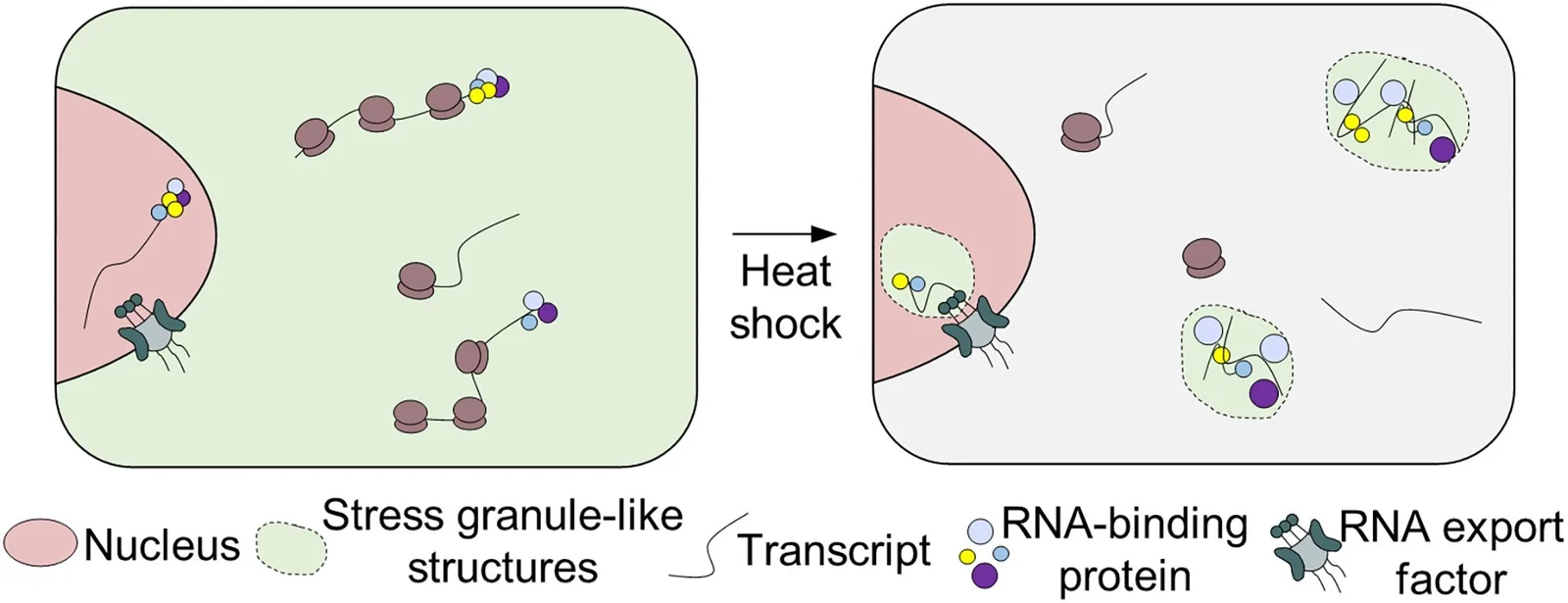

## Introduction

Eukaryotic cells employ sophisticated hierarchical systems to withstand diverse environmental stresses [1]. In response to stresses, they reprogram gene expression and modulate key biological processes, including RNA splicing, nuclear RNA export, and translation [2–4]. Besides these canonical responses, cells often form RNA-protein condensates known as stress granules (SGs) [5, 6], which arise when translation initiation is halted, causing polysome disassembly and accumulation of nontranslating messenger RNAs (mRNAs) in the cytoplasm [7, 8]. SG formation is facilitated by liquid–liquid phase separation (LLPS), sequestering RNAs and RNA-binding proteins into distinct compartments comprising stable cores and a surrounding shell [9]. SG cores are made up of many proteins, with certain RNA-binding proteins such as poly(A)-binding protein Pab1, recognized as SG markers. Dysregulation of SG dynamics has been linked to human diseases, including cancers and neurodegenerative disorders [10, 11], yet, their molecular composition and regulatory mechanisms remain incompletely understood. Although SGs have been extensively investigated in *Saccharomyces cerevisiae* [9, 12], our understanding of SG biology in filamentous fungi and the vast majority of fungal species remains limited.

Nuclear RNA export, another stress-responsive process, selectively controls gene expression under adverse conditions. Eukaryotic mRNA transcribed by RNA polymerase II undergoes a series of modifications, 5′ end capping, splicing, and 3′ end polyadenylation. Concurrently, nuclear RNA-binding proteins associate with the mRNA as part of the mRNA ribonucleoprotein (mRNP) biogenesis. Among the mRNP components, the mRNA export factor Yra1 in *S. cerevisiae* (ALYREF in metazoans) enables the mRNP to acquire export competence [13, 14]. Yra1 is a component of the transcript-export (TREX) complex, which is specifically recruited to transcriptionally active genes and couples nuclear mRNA export [15]. Upon TREX complex binding to a target mRNA, Yra1 facilitates interactions with the export receptor Mex67-Mtr2, which mediates mRNP transport through the nuclear pore to the cytoplasmic side [14, 16]. Notably, Yra1 predominantly localizes, together with other mRNA export factors, to nuclear speckles—sub-nuclear structures [17] that serve as central hubs for regulating nuclear RNA export.

The filamentous ascomycete *Fusarium graminearum* is a causal agent of wheat scab. *Fusarium graminearum* has been recognized as a model fungus for fungal genetics due to its advantages: high efficiency of gene knockout and a well-established outcrossing system. Systematic genetic research on *F. graminearum* has produced a wealth of genetic resources. To date, a large number of gene knockout mutants have been constructed, including those targeting transcription factors [18], kinases [19], phosphatases [20], and G-protein coupled receptors [21]. Leveraging the extensive genetic resources available for *F. graminearum* provides an opportunity to extend current knowledge of SGs or SG-like structures beyond yeast systems and to explore their biology in filamentous fungi. Here, we delineate the molecular architecture of *F. graminearum* SG-like structures in a heat shock-specific manner. Our study seeks to pave the way for future investigations into the complex mechanisms and roles of stress-induced condensates across eukaryotic organisms beyond fungi.

## Materials and methods

### Fungal strains and culture conditions

The *F. graminearum* wild-type strain Z-3639 [22] and its derivative mutants were routinely grown on complete medium (CM) agar plates (2 g NaNO_3_, 2.5 g peptone, 1 g yeast extract, 30 g sucrose, 1 g KH_2_PO_4_, 0.5 g MgSO_4_•7H_2_O, 0.5 g KCl, 0.2 ml trace element solution, 10 ml vitamin stock solution, 20 g agar, and 1 l water) at 25°C. Conidia of the fungal strains were harvested from 5-day-old carboxymethyl cellulose (15 g carboxymethyl cellulose, 1 g yeast extract, 0.5 g MgSO_4_•7H_2_O, 1 g NH_4_NO_3_, 1 g KH_2_PO_4_, and 1 l water) cultures. For stress tests, conidia and mycelia were incubated in liquid CM containing 10 mM H_2_O_2_ (oxidative stress), 1 M KCl (osmotic stress), 15% ethanol (ethanol stress), 5 µg/ml fludioxonil, or 2.5 µg/ml tebuconazole. For carbon starvation, minimal medium (MM) without a carbon source (2 g NaNO_3_, 1 g KH_2_PO_4_, 0.5 g MgSO_4_•7H_2_O, 0.5 g KCl, 0.2 ml trace element solution, and 1 l water) was used. For mycotoxin biosynthesis induction, starch-glutamate medium (SG medium, for zearalenone induction) or MM supplemented with 5 mM agmatine (MMA medium) were used.

### Generation of fluorescence-labeled strains

To construct the ectopic expression vectors, the open reading frames (ORFs), along with their native promoter regions, were amplified using the primers listed in Supplementary Table S1. The resulting PCR products were individually co-transformed with *XhoI*-digested pDL2 or pDL2/RFP vector into the *S. cerevisiae* strain PJ69-4A using the yeast gap repair approach [23]. Recombinant plasmids were extracted from the transformed yeast cells and subsequently transferred into *Escherichia coli* DH10B cells. Each final fusion construct was confirmed via PCR amplification and sequencing to verify its integrity. Thereafter, the resulting vectors were transformed into fungal protoplasts according to the PEG-mediated transformation protocol [24]. The truncated FgPab1-GFP construct (FgPab1^Δ488–671^-GFP) was generated through the same strategy.

### Fluorescent protein synthesis analysis

Protein synthesis was measured using a Click-iT^TM^ Plus OPP Alexa Fluor^TM^ 488 Protein Synthesis Assay Kit (Thermo Fisher Scientific, Waltham, MA, USA) according to the manufacturer’s instructions. Briefly, conidia were incubated with OPP in the presence or absence of heat shock treatment, followed by fixation, permeabilization, and click-labeling with Alexa Fluor^TM^ 488 azide. Fluorescence intensity was quantified using a DM6 B microscope and LAS X software.

### Generation of knockout mutant strains

Gene knockout mutants were generated through the double-joint PCR strategy [25]. Briefly, the upstream and downstream flanking sequences of the target ORFs were amplified with the primer pairs 5F/5R and 3F/3R, respectively. The resulting amplicons were fused with the geneticin resistance cassette obtained from pII99 plasmid [26]. The final DNA fragments were amplified using the nested primers to split the marker genes and were then subjected to fungal transformation. After transforming the *F. graminearum* wild-type strain Z-3639, all transformants were primarily screened by diagnostic PCR and further verified via Southern blot hybridization.

### Plant infection and mycotoxin production assay

Two-day-old seedlings of the wheat cultivar Eunpa were used for the plant infection assay as described previously [21]. The top 2 mm portion of the coleoptiles was cut off and inoculated with 2 μl of conidial suspensions (1 × 10^6^ conidia/ml) over the wounded sites. These seedlings were then grown at 25°C in a moisture chamber. Necrotic lesions on wheat seedlings were measured at 7 days after inoculation. To examine whether SG-like structures are observed during plant infection, wheat coleoptiles inoculated with transformants expressing FgPab1-GFP were sampled at 24 or 96 h after inoculation and observed using a DM6 B microscope (Leica Microsystems, Wetzlar, Germany) equipped with a Leica DMC6200 camera.

For mycotoxin production assay, 1.5 g of rice grains were placed in 8 ml vials and incubated with *F. graminearum* strains at 25°C for 3 weeks. The rice cultures were ground and mixed vigorously with 6 ml of 84% acetonitrile (ACN) for 30 min. After centrifugation at 1500 revolutions per minute for 5 min, the supernatant was filtered through a 0.45 μm syringe filter. Mycotoxin analysis was performed using reverse-phase high performance liquid chromatography (HPLC) on a Prominence HPLC system (Shimadzu, Kyoto, Japan) equipped with a C18 column, following previously described methods with minor modifications [27]. Zearalenone was quantified using a mobile phase of 70% aqueous methanol at a flow rate of 1 ml/min. Deoxynivalenol was analyzed with a mobile phase containing 10% ACN at the same flow rate, employing the following gradient program: hold at 10% ACN for 11 min; increase linearly to 30% ACN by 12 min; decrease back to 10% ACN by 18 min; maintain for 17 min for column re-equilibration; then proceed to the next injection, for a total runtime of 35 min. Both zearalenone and deoxynivalenol were detected at 235 nm using diode-array detection.

### Sexual crosses

Sexual reproduction was induced by incubating *F. graminearum* strains on carrot agar plates at 25°C for 5 days. For self-fertilization, aerial hyphae grown on carrot agar were mock-fertilized with sterile 2.5% Tween 60 solution. For outcrossing, the heterothallic female strain (Δ*mat1-1*::hH1-RFP) [28] was fertilized with 1 ml of conidial suspension from the male strain at 5 days post-inoculation on carrot agar. All fertilized cultures were then incubated at 25°C for 8 days under near-ultraviolet light (wavelength: 365 nm; Sankyo Denki, Tokyo, Japan).

### Isolation of SG-like structures

The isolation of SG-like structures was conducted following previously described protocols with minor modifications [9, 12]. Briefly, *F. graminearum* strains expressing FgPab1-GFP were cultivated in liquid CM and subjected to heat shock for 15 min at 42°C. After heat shock, mycelia were harvested and immediately ground to a fine powder in liquid nitrogen. The powder samples were suspended in 1 ml SG lysis buffer [50 mM Tris–HCl, pH 7.4; 2 mM magnesium acetate; 100 mM potassium acetate; 0.5 mM dithiothreitol; 50 μg/ml Heparin; 0.5% NP-40; 0.02% Antifoam B; mini ethylenediaminetetraacetic acid (EDTA)-free protease inhibitor tablet; and 1 U/μl RNase Plus RNase Inhibitor] and sonicated using an ultrasonic homogenizer. The resulting lysates were centrifuged at 16 000 × *g* for 20 min at 4°C. The supernatants were carefully transferred to new microcentrifuge tubes and centrifuged at 18 000 × *g* for 10 min at 4°C. The sediment containing SG-like structures was then resuspended in 1 ml SG lysis buffer and centrifuged again at 14 000 × *g* for 10 min at 4°C. The resulting pellet was resuspended in 50 μl SG lysis buffer and spun at 850 × *g* for 2 min at 4°C to obtain the supernatant, referred to as the enriched fraction.

The enriched fraction was further purified using anti-GFP monoclonal antibody (mAb)-conjugated magnetic beads (ABclonal, AE079) to obtain the SG-like fraction (SG fraction). The SG fraction immunoprecipitated with anti-GFP magnetic beads was washed with two types of wash buffers: wash buffer 1 (SG lysis buffer supplemented with 2 M urea) and wash buffer 2 (SG lysis buffer supplemented with 300 mM potassium acetate).

### RNA sequencing

Total RNA was extracted from the mycelial powder, enriched fraction, and SG fraction of control and heat shock samples using an Easy-Spin Total RNA Extraction Kit (iNtRON Biotechnology, Seongnam, Republic of Korea) for RNA-seq analysis. Total RNA from each sample was used for RNA-seq library construction with a TruSeq Stranded Total RNA Library Prep Human/Mouse/Rat Kit, following the manufacturer’s protocol. To obtain a comprehensive profile of SG-associated transcripts, ribosomal RNA was not removed during library preparation. The RNA-seq libraries were sequenced on an Illumina NovaSeq X system in 101 bp paired-end read mode.

The quality of raw reads was assessed using the FastQC tool. Low-quality reads and Illumina adapters were trimmed with Trimmomatic [29] using default settings. The processed RNA-seq reads were aligned to the *F. graminearum* reference genome (available at FgBase [30] and NCBI Genome Database) by HISAT2 [31] and visualized with the Integrative Genomics Viewer (IGV) tool. Gene-level read counts were obtained with featureCounts [32], assigning fractional counts of 1/n to multimapped reads, where n represents the total number of alignments for a given read. Differentially expressed transcripts (DETs; *P* <.01 and fold change of ≥2) were identified using the edgeR [33] package in R. Gene Ontology (GO) analysis of DETs was performed with the DAVID [34] functional annotation tool to obtain enriched GO terms.

### Reverse transcription PCR

Total RNA was used for first-strand complementary DNA (cDNA) synthesis with SuperScript III First-Strand Synthesis System (Invitrogen, Carlsbad, CA, USA), employing an Oligo(dT)_20_ primer. The synthesized cDNA was used for PCR validation of introns in each gene using the corresponding primers. For control, the endogenous housekeeping gene ubiquitin C-terminal hydrolase (*UBH1*) was used.

### Proteomic analysis

For proteomic analysis, the SG fraction was used to identify SG-like structure constituents. The samples were loaded onto sodium dodecyl sulfate–polyacrylamide (SDS–PAGE) gels and separated through electrophoresis. The bands of interest were excised and digested with trypsin. Tryptic peptides were analyzed using a Q Exactive system coupled to an EASY-nLC-1000 nanoflow liquid chromatography system. Peptide samples were first concentrated on an Acclaim PepMap 100 C18 column (75 μm × 2 cm) and subsequently resolved on an EASYSpray analytical column (75 μm × 15 cm; particle size ≤ 3 μm, pore size 100 Å, PepMap^TM^ RSLC C18). Chromatographic separation was achieved with a 90 min gradient from 5% to 40% solvent B (0.1% formic acid in ACN) at a flow rate of 300 nl/min and a maintained column temperature of 35°C. Peptides were ionized at 2 kV and analyzed in a full MS scan mode (m/z 400–2000) at a resolution of 70 000 full width at half-maximum. The instrument then performed data-dependent higher-energy collisional dissociation tandem mass spectrometry (HCD MS/MS) acquisition on the top 10 most intense precursor ions, with a resolution of 17 500, a loop count of 10, an isolation width of m/z 2.0, and a normalized collision energy of 27.

Raw mass spectrometry data were processed in Proteome Discoverer (v2.4.1.15) against the *F. graminearum* protein database. Searches were conducted using the Sequest HS algorithm with trypsin specificity, allowing up to two missed cleavages, a precursor mass tolerance of 10 ppm, and a fragment mass tolerance of 0.02 Da. Carbamidomethylation of cysteine was defined as a fixed modification, while methionine oxidation was considered a variable modification. Peptide and protein identifications were validated and quantified in Scaffold (v5.0.1), applying SEQUEST XCorr score thresholds of ≥ 1.8 (charge 1), ≥ 2.5 (charge 2), and ≥ 3.5 (charge 3 and 4). Semiquantitative estimates were obtained based on total spectral counts.

The Enriched/Total ratio was calculated by comparing protein abundance in the SG-like structure-enriched fraction with that in the corresponding whole-cell lysate prepared under heat shock conditions. Prior to downstream comparison, total spectral counts were normalized across samples. An Enriched/Total ratio of ≥ 0.7 was empirically selected based on proteins with independent evidence of SG association, including previously reported SG-associated proteins and proteins validated by co-localization analyses in this study. This threshold was used as a practical criterion for identifying putative SG-associated candidates rather than as a definitive biological cutoff.

### Western blot and co-immunoprecipitation

Total proteins were extracted from fungal mycelia and separated on 8% SDS–PAGE gels and transferred to nitrocellulose membranes using the Trans-Blot Turbo Transfer System. The membranes were incubated with blocking buffer (5% skim milk with TBS-T [20 mM Tris–HCl, pH 7.6; 140 mM NaCl; 0.1% Tween-20]) overnight at 4°C to block nonspecific antibody binding. The proteins were detected using anti-GFP (GenScript, A01388), anti-RFP (Abcam, ab124754), anti-eIF2α (Cell Signaling Technology, 9722), anti-histone H3 (Abcam, ab1791), or anti-GAPDH (ABclonal, A19056) primary antibodies and their corresponding secondary antibodies (Abcam, ab6721) at 1:4000 dilution with a Chemi-Doc imaging system (Bio-Rad, Hercules, CA, USA).

For co-immunoprecipitation (co-IP) assay, SG-like structure-enriched fractions were incubated with anti-GFP magnetic beads overnight at 4°C. Both total protein extracts and proteins eluted from the beads were separated by SDS–PAGE and subsequently analyzed by western blotting.

### Nuclei isolation

A total of 2 g of mycelia was finely ground into a powder using a mortar and pestle with liquid nitrogen. The resulting powder was incubated in 5 ml lysis buffer (20 mM Tris–HCl, pH 7.5; 20 mM KCl; 2 mM EDTA; 2.5 mM MgCl_2_; 25% glycerol; 250 mM sucrose; and 5 mM dithiothreitol) for 15 min on ice. The homogenate was subsequently filtered through a cell strainer to remove cellular debris, and the filtrate was centrifuged at 1500 × *g* for 10 min at 4°C. The pellet containing the cell nuclei was then resuspended in 1 ml of NRBT buffer (20 mM Tris–HCl, pH 7.5; 2.5 mM MgCl_2_; 25% glycerol; and 0.2% Triton X-100) and centrifuged at 1500 × *g* for 10 min at 4°C. This step was repeated four times to ensure thorough purification of the nuclei. After complete removal of the NRBT buffer, the sediment was resuspended in 500 μl of NRB2 buffer (20 mM Tris–HCl, pH 7.5; 10 mM MgCl_2_; 250 mM sucrose; 0.5% Triton X-100; and 5 mM β-mercaptoethanol). The suspension was carefully layered over 500 μl of NRB3 buffer (20 mM Tris–HCl, pH 7.5; 10 mM MgCl_2_; 1.7 M sucrose; 0.5% Triton X-100; and 5 mM β-mercaptoethanol) and centrifuged at 16 000 × *g* for 45 min at 4°C. The final nuclear pellet was resuspended in 400 μl lysis buffer and used for nuclear RNA-seq library preparation.

### Microscopic observations

For SG-like structure observation, *F. graminearum* cells were imaged using a DM6 B microscope (Leica Microsystems, Wetzlar, Germany) equipped with a Leica DMC6200 camera. A Leica HC PL FLUOTAR 40×/0.80 objective was used for routine fluorescence imaging, whereas a Leica N PLAN 100×/1.25 oil immersion objective was used for high-magnification observation of SG-like structures. Images were acquired using a fixed exposure time of 500 ms under identical imaging conditions. Representative images and quantitative analyses were performed using single focal-plane fluorescence images. No Z-stack acquisition or projection was performed. For time-lapse imaging analysis, *F. graminearum* specimens were mounted on a hot plate (DAIHAN Scientific, Wonju, Republic of Korea) maintained at 42°C, and images were acquired at 15-s intervals.

*Fusarium graminearum* nuclei were stained by mounting samples in Hoechst 33258 solution (0.5 μg/ml), followed by incubation in the dark at room temperature for 15 min. After washing with phosphate-buffered saline, fluorescence images were captured.

### Quantification and statistical analysis

Image analysis was performed using Leica LAS X software. Fluorescence intensity in the Click-iT OPP assay was quantified by measuring the mean fluorescence intensity using the measurement tools in LAS X under identical image acquisition settings. SG-like structures were quantified by manual counting from single focal-plane fluorescence images using the same scoring criteria for all samples.

Statistical analysis was carried out in Microsoft Excel or GraphPad Prism. Statistical comparisons among multiple groups were conducted using one-way analysis of variance (ANOVA). Comparisons between two groups were performed using a two-tailed unpaired Student’s *t*-test. Differences with *P*-values < 0.05 were considered statistically significant.

## Results

### Heat shock induces SG-like structure formation

**Figure 1.**
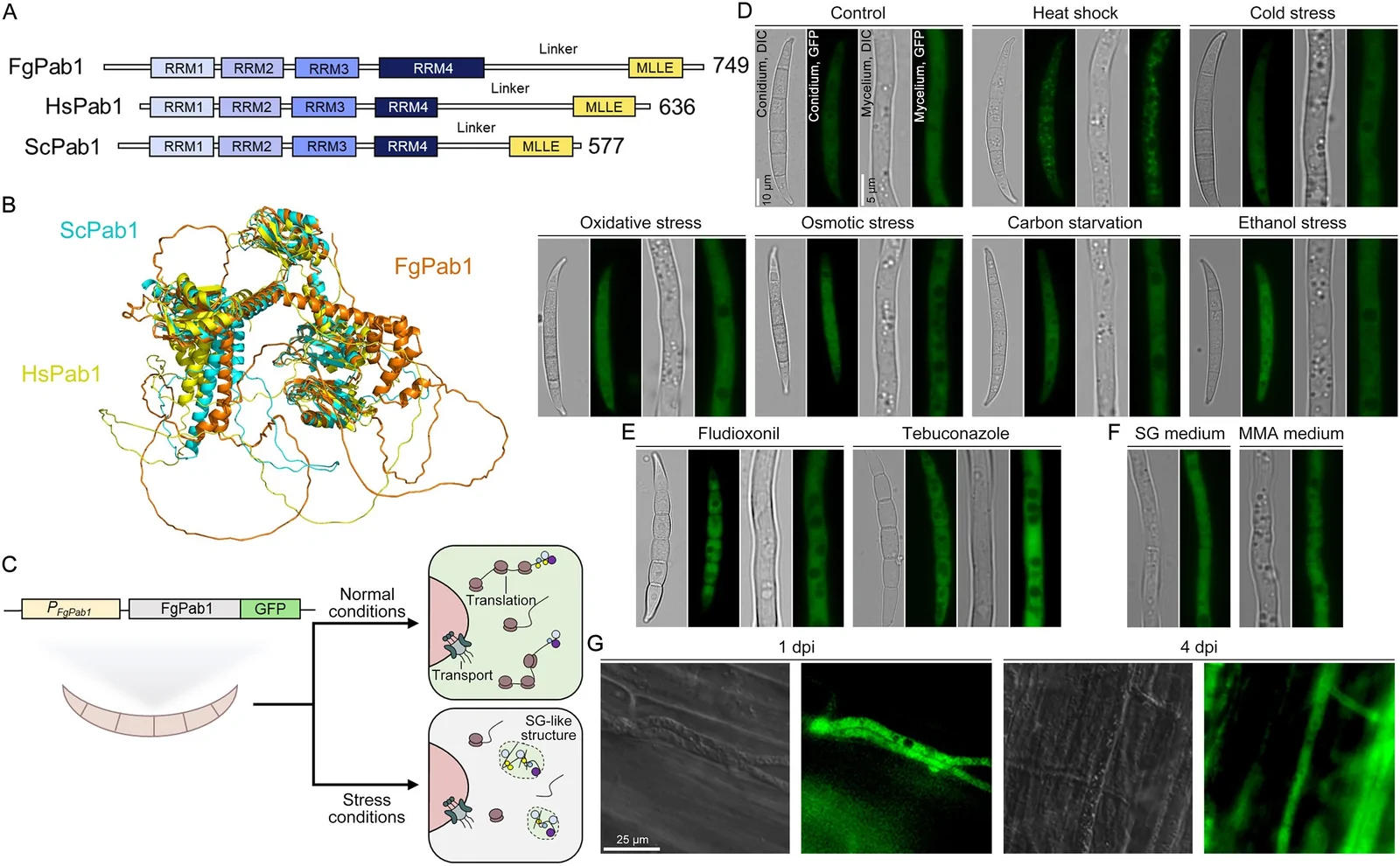
Heat shock induces FgPab1-GFP foci formation in *F. graminearum*. (**A**) Domain architectures of FgPab1 and its orthologs in humans (*H. sapiens*, Hs) and yeast (*S. cerevisiae*, Sc). (**B**) An AlphaFold model depicting structural overlap analysis of Pab1 orthologous proteins. (**C**) A schematic illustration showing SG-like structure formation and the transformant ectopically expressing FgPab1-GFP under its native promoter. (**D–G**) Representative microscopic images of cells expressing FgPab1-GFP subjected to the denoted stresses. Stress treatments include (**D**) culture condition stresses, (**E**) fungicide stresses, (**F**) mycotoxin biosynthesis induction, and (**G**) plant infection stress.

Across eukaryotic species, from humans to yeast, Pab1 has been widely recognized as a marker protein for SGs [9]. In *F. graminearum*, the FGRAMPH1_01G09919 gene encodes the putative ortholog of Pab1. Domain architecture analysis revealed that FgPab1 contains four RNA recognition motifs and a C-terminal MLLE domain required for protein-protein interactions (Fig. 1A). The predicted structure of FgPab1 closely resembled its counterparts in *S. cerevisiae* and *Homo sapiens* (Fig. 1B), suggesting that *F. graminearum* Pab1 likely has conserved features, including stress-induced condensation.

To investigate whether *F. graminearum* forms FgPab1-positive assemblies under different stress conditions, we generated a strain ectopically expressing FgPab1-GFP under its native promoter (Fig. 1C) and exposed it to various stresses. Microscopic observations revealed the formation of FgPab1-GFP foci in both conidia and mycelia after heat shock (42°C for 15 min) (Fig. 1D). Because these foci were specifically induced by heat shock and shared morphological characteristics with canonical SGs, we hereafter refer to them as SG-like structures. However, other stress conditions—such as nutrient starvation, oxidative stress, osmotic stress, fungicide treatment, mycotoxin biosynthesis induction, and plant infection—did not induce FgPab1-GFP foci formation (Fig. 1D–G). Therefore, these findings suggest that SG-like structures are formed specifically in response to heat shock in *F. graminearum*.

### Heat shock-induced FgPab1-GFP foci exhibit dynamic assembly

**Figure 2.**
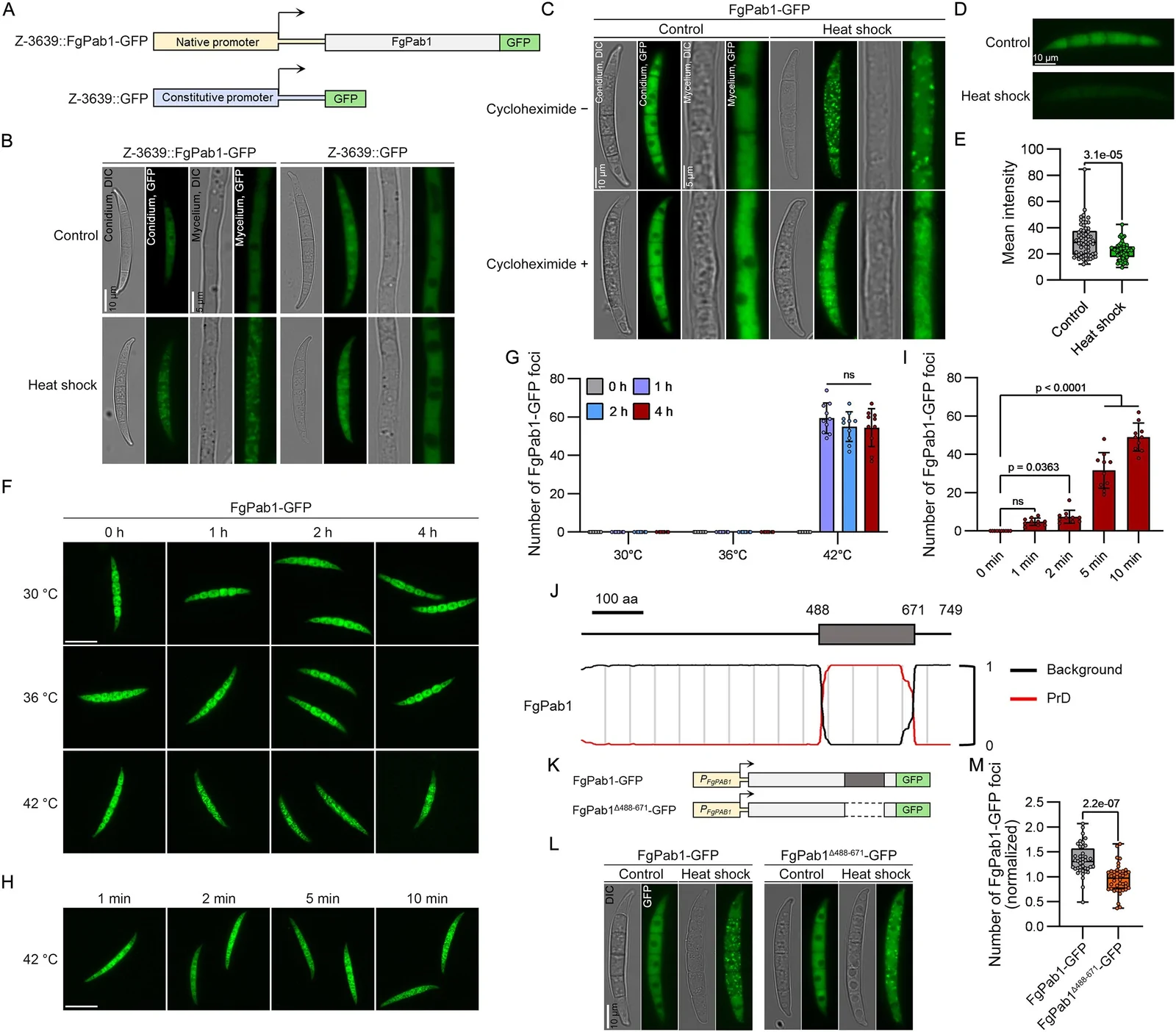
Heat shock-induced FgPab1-GFP foci exhibit hallmark properties and dynamic assembly. (**A, B**) Comparison of the behavior of FgPab1-GFP and free GFP under heat shock conditions. Constructs used to assess GFP foci formation in response to heat shock are depicted in panel (A), and representative microscopy images are shown in panel (B). (**C**) Sensitivity of heat shock-induced FgPab1-GFP foci to cycloheximide. *Fusarium graminearum* cultures were grown in the presence of cycloheximide (100 μg/ml) for 30 min prior to heat shock treatment. (**D**) Fluorescent protein synthesis assay. Newly synthesized proteins were visualized as green fluorescent signals. (**E**) Quantification of the mean fluorescence intensity in transverse sections of each conidium (*n* = 60) from panel (D). (**F**) Hourly time course analysis of heat shock-induced FgPab1-GFP foci at the indicated temperatures. Scale bar = 25 μm. (**G**) Quantification of FgPab1-GFP foci per conidium from panel (F). (**H**) Minute-scale time course analysis of heat shock-induced FgPab1-GFP foci. Scale bar = 25 μm. (**I**) Quantification of FgPab1-GFP foci per conidium from panel (H). (**J**) Identification of the prion-like domain (PrD) in FgPab1 via PLAAC analysis. (**K**) Schematic of PrD-truncated FgPab1 constructs. (**L**) Microscopy images of SG-like structure formation in transformants expressing the PrD-truncated construct shown in panel (K). (**M**) Quantification of FgPab1-GFP foci per conidium (*n* = 40) from panel (**L**). For panels (E) and (M), statistical significance was determined using Student’s *t*-test. For panels (G) and (I), data represent the mean and standard deviation from independent replicates (*n* = 10). Statistical significance was determined by one-way ANOVA followed by Tukey’s multiple comparisons test.

Because high temperature can induce nonspecific protein aggregation [35], we first compared the behavior of FgPab1-GFP with that of free GFP. A control strain expressing free GFP under a constitutive promoter (Fig. 2A) showed no foci formation after heat shock, whereas FgPab1-GFP foci were clearly visible (Fig. 2B). Additionally, we observed that heat shock-induced FgPab1-GFP foci were sensitive to cycloheximide (Fig. 2C), an inhibitor of translation elongation that prevents SG assembly [36, 37]. Given that SGs form when translation initiation is impaired and global protein synthesis is reduced [5], we examined bulk translation levels under heat shock conditions. Consistent with formation of FgPab1-GFP foci, bulk translation was significantly reduced in heat-shocked cells compared with unstressed controls (Fig. 2D and E). Further, FgPab1-GFP foci co-localized with RFP-labeled foci of several previously reported SG-associated proteins (Supplementary Table S2) [9, 38–40], including FgBre5, FgeIF4E, FgUbp3, FgNgr1, FgPbp1, FgLsm7, and FgPub1, under heat shock conditions (Supplementary Fig. S1). Taken together, these findings support the interpretation that the heat shock-induced FgPab1-GFP foci share traits commonly associated with SG-like structures.

We next examined temporal dynamics of SG-like structure formation. Prolonged incubation at 42°C caused no significant change in the number of FgPab1-GFP foci per conidium (Fig. 2F and G). Minute-scale observation revealed that FgPab1-GFP foci became visible within 2 min of heat shock and gradually increased over time (Fig. 2H and I, and Supplementary Movie S1).

Since LLPS has been implicated in SG formation [6, 41], we wondered whether FgPab1 contains sequence features that may facilitate the formation of heat shock-induced SG-like structures. PLAAC analysis [42] identified a prion-like domain (PrD; 488–671 aa) at the C-terminal region (Fig. 2J). To assess its contribution, we generated a PrD-truncated construct (FgPab1^Δ488–671^-GFP) under the control of its native promoter (Fig. 2K). Interestingly, heat shock-induced FgPab1-GFP foci were markedly reduced in the truncated construct compared with those formed by the full-length protein (Fig. 2L and M).

### Heat shock-dependent SG-like structure formation is not affected by genetic perturbation

To determine the role of other SG-associated components in *F. graminearum* (Supplementary Table S2), we generated knockout mutants. Knockout mutants were obtained for all genes except *FgPAB1, FgeIF4E*, and *FgLSM7*, which could not be deleted after three transformation attempts, suggesting essentiality (Supplementary Fig. S2A–E). Although some knockout mutants exhibited phenotypic differences, including vegetative growth and sexual development (Supplementary Figs S3A–E and S4), these differences varied among the mutants and did not correlate with their ability to form heat shock-induced SG-like structures. Upon heat shock, robust SG-like structure assembly was observed in all mutants (Supplementary Fig. S5A and B).

### Longer transcripts are preferentially enriched in heat shock-induced SG-like structures

**Figure 3.**
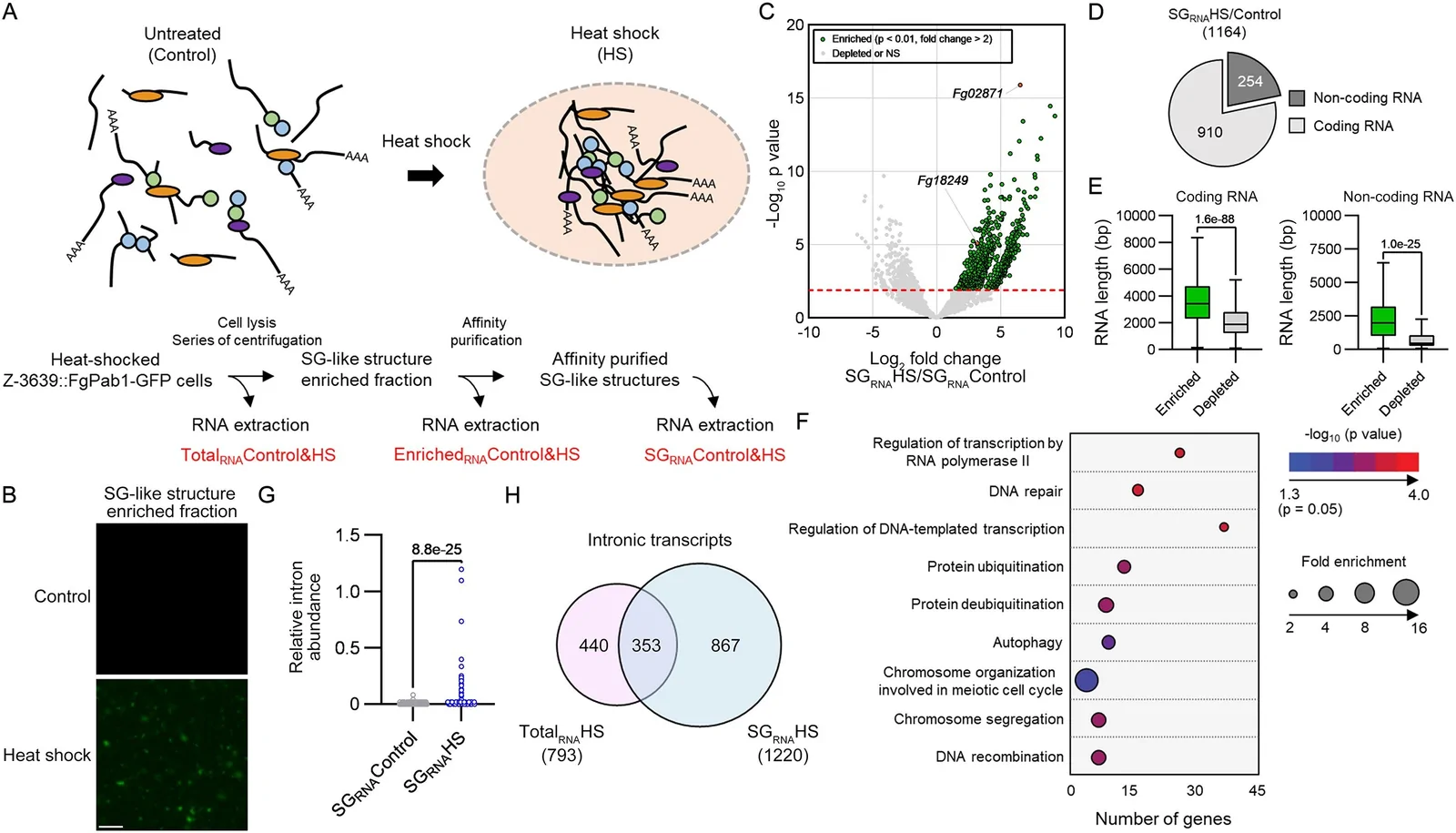
Transcriptomic profiling defines the RNA landscape of SG-like structures. (**A**) Schematic of the workflow for isolation of SG-like structures followed by transcriptome profiling. (**B**) SG-like structure-enriched fraction from FgPab1-GFP expressing cells. Scale bar = 5 μm. (**C**) A volcano plot of DETs comparing SG_RNA_HS and SG_RNA_Control. Transcripts with an adjusted *P*-value < 0.01 and log_2_ fold change > 1 are highlighted and designated as SG-like structure-specific transcripts. (**D**) Charts showing the number of noncoding and coding RNAs enriched in the DETs from panel (C). (**E**) Box plots depicting total transcript lengths of coding and noncoding transcripts. (**F**) GO analysis of the SG-like structure-specific transcripts. Dot color represents *P*-value, and dot size indicates fold enrichment. (**G**) Comparison of relative intron abundance between SG_RNA_Control and SG_RNA_HS. Average read counts mapped to each intronic region are compared (*n* = 8546). (**H**) Overlap of intronic transcripts enriched in Total_RNA_HS and SG_RNA_HS. For panels (E) and (G), statistical significance was estimated based on Student’s *t*-test.

To define the RNA composition of SG-like structures in *F. graminearum*, we performed RNA-seq after purification of SG-like structures from cells subjected to heat shock (42°C for 15 min) or control conditions (25°C) (Fig. 3A). FgPab1-GFP foci remained detectable throughout the purification process in heat shock-treated cell lysates (Fig. 3B). Total_RNA_ (extracted directly from *F. graminearum* cells), Enriched_RNA_ (extracted from SG-like structure-enriched fraction), and SG_RNA_ (extracted from SG-like structures) were prepared to validate enrichment specificity (Supplementary Table S3 and Supplementary Fig. S6A). Heat-shock-induced transcripts were highly concordant between Enriched_RNA_HS/Control and SG_RNA_HS/Control (Supplementary Fig. S6B and C), confirming selective enrichment.

To explore the transcriptome of SG-like structures, we delved into the SG_RNA_HS/Control dataset and defined DETs as SG-like structure-specific transcripts. We identified 1164 DETs (≥ 2-fold change, *P* < 0.01) (Fig. 3C), comprising 910 coding RNAs and 254 noncoding RNAs (Fig. 3D). Both coding and noncoding DETs were significantly longer than average transcripts (Fig. 3E). GO analysis revealed enrichment in functions related to DNA repair, protein deubiquitination/ubiquitination, autophagy, and transcriptional regulation (Fig. 3F). Taken together, these results indicate that SG-like structure-specific transcripts are characterized by longer lengths and distinct functional roles.

### Heat shock leads to accumulation of intronic RNA in SG-like structures

To gain deeper insights into the SG-like structure transcriptome, we further analyzed its biophysical features. These transcripts showed significantly higher intron abundance compared to controls (Fig. 3G), with no significant differences in 5′- or 3′-untranslated region (Supplementary Fig. S6D and E). We next examined whether intron accumulation specific to SG-like structures is distinct from that observed in the total RNA population. Likewise, heat shock led to increased intron abundance in the total RNA-seq data (Supplementary Fig. S6F). Of the 1220 intronic transcripts specifically enriched in SG-like structures, however, only 353 overlapped with those enriched in the total RNA pools, indicating a partial overlap between the two datasets (Fig. 3H). Further analysis revealed that SG-like structure-enriched intronic transcripts did not exhibit any prominent characteristics (Supplementary Fig. S7A–C). These results suggest that heat shock broadly promotes intron abundance, while a subset of these intronic transcripts is selectively recruited to SG-like structures.

### SG-like structures form in the nucleus under heat shock

**Figure 4.**
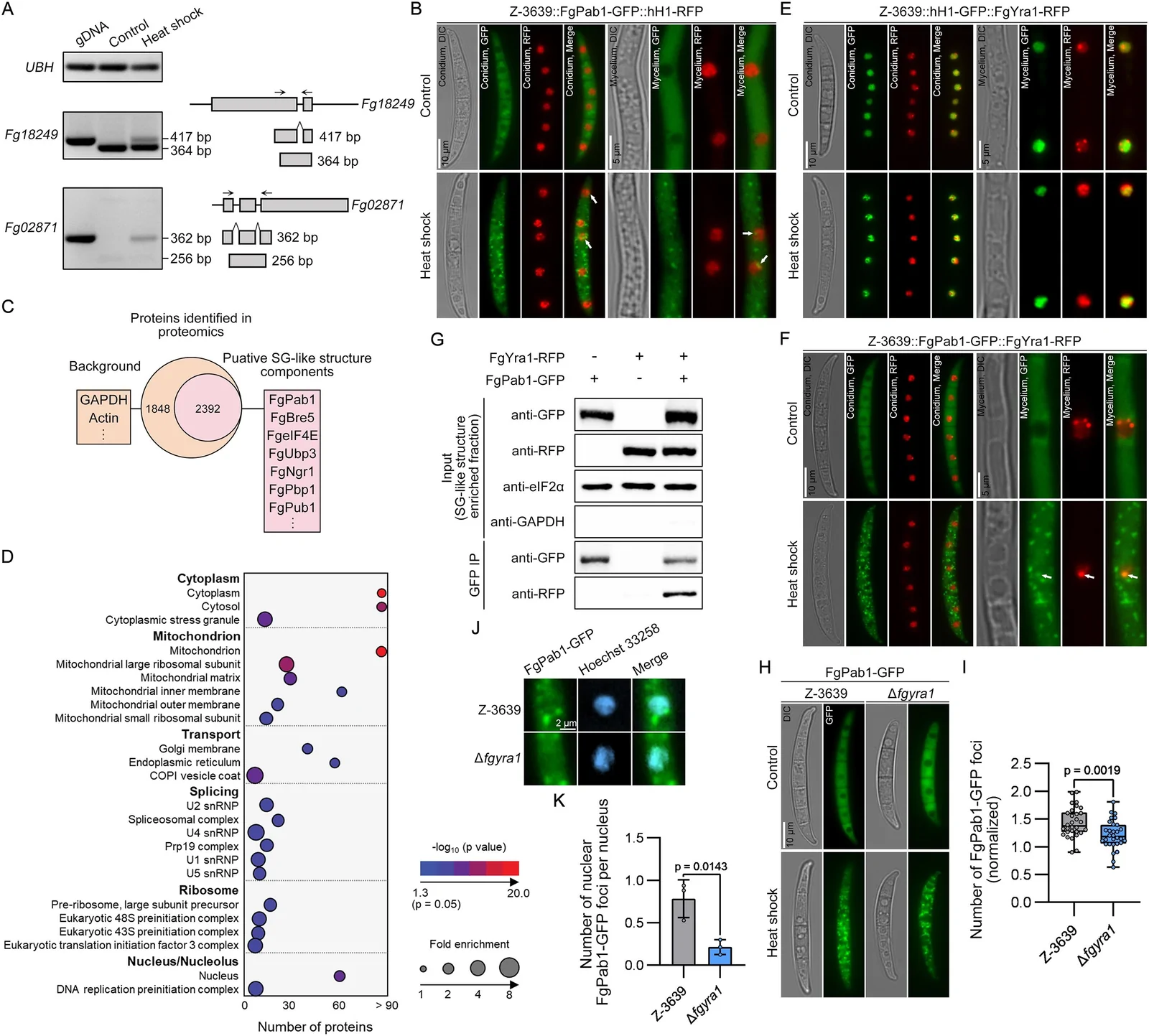
SG-like structures sequester FgYra1, or vice versa. (**A**) RT-PCR analysis of intron retention in heat shock-treated cells. The expected positions of RT-PCR products corresponding to spliced and intron-retained transcripts are illustrated to the right of each gel image. Genomic DNA was included as a positive control for intron-containing amplicons. Control indicates unstressed conditions. *UBH*, ubiquitin C-terminal hydrolase. (**B**) Fluorescence microscopy of *F. graminearum* co-expressing FgPab1-GFP and histone H1-RFP following heat shock treatment. (**C**) Venn diagram showing proteins classified according to their relative enrichment in the proteomics datasets. Proteins were classified as putative SG-like structure components or background proteins based on their enrichment during purification of SG-like structures. (**D**) Dot plot showing enriched GO terms among the putative SG-like structure components identified in panel (C). (**E, F**) Fluorescence microscopy of *F. graminearum* co-expressing (**E**) histone H1-GFP/FgYra1-RFP and (**F**) FgPab1-GFP/FgYra1-RFP following heat shock treatment. (**G**) Co-IP assay of FgPab1-GFP and FgYra1-RFP during heat shock conditions. SG-like structure-enriched fractions were used as input, and eIF2α served as a loading control. (**H**) Representative images of heat shock-induced FgPab1-GFP foci formation in the wild-type and Δ*fgyra1* mutant strains. (**I**) Quantification of FgPab1-GFP foci per conidium (*n* = 30) from panel (H). To account for differences in conidial length between the wild-type and mutant strains, the number of FgPab1-GFP foci was normalized to conidial length. (**J**) Representative images of nuclear FgPab1-GFP foci in the wild-type and Δ*fgyra1* mutant strains. (**K**) Quantification of the number of nuclear FgPab1-GFP foci per nucleus from panel (**J**). Data represent the mean and standard deviation from biological replicates (*n* = 3) with at least 50 nuclei examined in each replicate. For panels (I) and (K), statistical significance was assessed based on Student’s *t*-test.

We next pursued the hypothesis that SG-like structures harbor intronic transcripts in a heat shock-specific manner. To test this, we first conducted reverse transcription PCR (RT-PCR) to validate intron retention in selected transcripts (FGRAMPH1_01G18249 and FGRAMPH1_01G02871) that showed the highest intron enrichment in our RNA-seq data. Notably, intron-retaining transcripts were detected exclusively in samples subjected to heat shock (Fig. 4A and Supplementary Fig. S8), suggesting that splicing patterns are altered in response to heat shock.

Because nonspliced mRNAs are typically sequestered in the nucleus, we reasoned that the accumulation of intronic transcripts in SG-like structures might indicate a functional association with nuclear RNA export. To investigate this, we examined SG-like structures with a focus on nuclear localization. Surprisingly, upon heat shock, FgPab1-GFP foci were observed not only in the cytoplasm but also within nuclei marked by histone H1-RFP (Fig. 4B). Moreover, several RFP-tagged SG-associated proteins also formed nuclear foci under heat shock conditions (Supplementary Fig. S9). These observations indicate that multiple SG-associated proteins exhibit stress-induced nuclear puncta, although they do not establish that these proteins co-localize with FgPab1 or occupy the same nuclear structures. These findings prompted us to further investigate a potential association between nuclear FgPab1-positive foci and nuclear RNA export.

To further explore this potential link, we carried out total proteomics and affinity purification-mass spectrometry (AP-MS) analyses using FgPab1-GFP as bait (Supplementary Fig. S10). Analysis of the proteomics datasets identified multiple SG-associated proteins (Fig. 4C and Supplementary Table S4), many of which remained associated with FgPab1-GFP both in cell lysates and *in vivo* (Supplementary Fig. S11). GO analysis of putative SG-like structure components revealed significant enrichment of cellular component terms related to the nucleus (Fig. 4D). Among these proteins, FgYra1 (FGRAMPH1_01G20577), an mRNA export factor, was identified as a putative component of SG-like structures. Consistent with previous findings [17, 43], FgYra1-RFP localized predominantly to nuclear speckles, a type of sub-nuclear structure (Fig. 4E).

We next examined whether heat shock induced the association of SG-like structures with nuclear speckles. To our surprise, upon heat shock, nuclear FgPab1-GFP foci co-localized with nuclear speckles in hyphae (Fig. 4F and Supplementary Fig. S12). Co-IP assays further supported an association between FgPab1 and FgYra1 under heat shock conditions (Fig. 4G). Additionally, deletion of *FgYRA1* resulted in a slight but reproducible reduction in both overall FgPab1-GFP foci formation (Fig. 4H and I), and nuclear FgPab1-GFP foci formation (Fig. 4J and K). Together, these results indicate that heat shock induces the formation of nuclear FgPab1 foci that physically associate with nuclear speckles. These observations suggest a potential relationship between nuclear FgPab1 foci and heat shock-associated changes in nuclear RNA dynamics.

### Heat shock alters nuclear transcript retention

**Figure 5.**
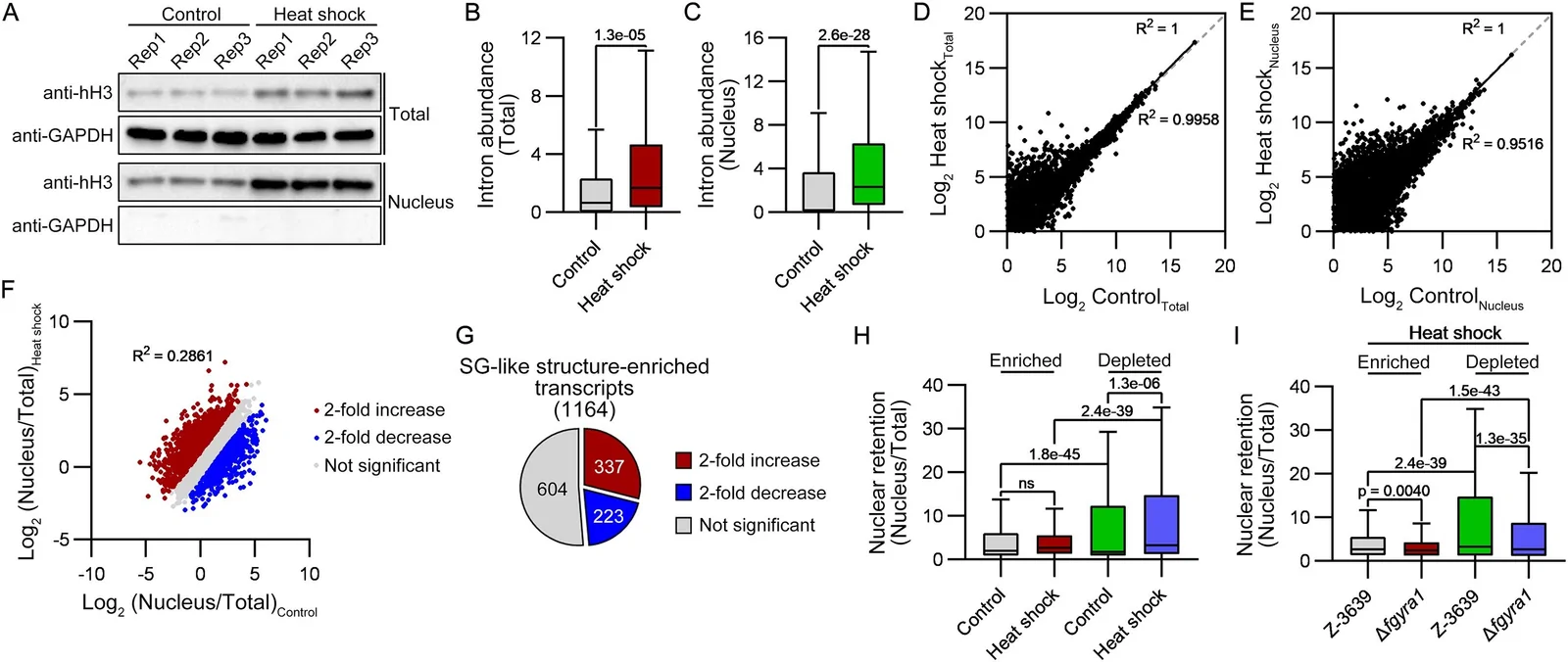
Heat shock alters nuclear transcript retention. (**A**) Western blot analysis confirming the purity of total and nuclear fractions. GAPDH and histone H3 were used as cytoplasmic and nuclear markers, respectively. (**B, C**) Box plots showing intronic read abundance under control and heat shock conditions. Data derived from total RNA-seq are shown in panel (B), and those from nuclear RNA-seq are shown in panel (C). (**D, E**) Scatter plots comparing transcript abundance (transcripts per million, TPM) between control and heat shock conditions. Data derived from total RNA-seq are shown in panel (D), whereas those from nuclear RNA-seq are shown in panel (E). (**F**) Relationship between nuclear retention scores (Nucleus/Total) under control and heat shock conditions. (**G**) Quantification of SG-like structure-enriched transcripts exhibiting significant changes in nuclear retention following heat shock treatment. (**H**) Box plots showing changes in nuclear retention of SG-like structure-enriched and SG-like structure-depleted transcripts under control and heat shock conditions. (**I**) Box plots depicting nuclear retention dynamics of SG-like structure-enriched and SG-like structure-depleted transcripts in the wild-type and Δ*fgyra1* strains under heat shock conditions. For panels (B), (C), (H), and (I), statistical significance was determined using Student’s *t*-test.

The selective sequestration of the nuclear RNA export factor FgYra1 into SG-like structures prompted us to propose that heat shock influences nuclear RNA export dynamics. To test this, we isolated nuclear fractions (Fig. 5A) and performed parallel RNA-seq analyses on nuclear and total RNA samples (Supplementary Table S3). Consistently, intron abundance significantly increased under heat shock conditions in both total and nuclear RNA-seq datasets (Fig. 5B and C), suggesting that heat shock induces the accumulation of intron-containing transcripts within the nucleus.

To further evaluate the impact of heat shock on RNA distribution, we compared transcript abundance between control and heat shock conditions using total and nuclear RNA-seq datasets. Transcript abundance was highly correlated between the two conditions in both total and nuclear RNA populations (Fig. 5D and E), indicating that heat shock exerts only a limited effect on overall transcript levels. In contrast, nuclear retention scores (Nucleus/Total ratio) were markedly altered following heat shock treatment (Fig. 5F). The weak correlation observed between control and heat shock conditions suggests that heat shock primarily affects the subcellular distribution of transcripts rather than global transcript abundance, thereby perturbing nuclear RNA export.

We next interrogated whether transcripts associated with SG-like structures exhibit distinct nuclear retention behaviors during heat shock. A substantial fraction of SG-like structure-enriched transcripts displayed significant changes in nuclear retention following heat shock treatment (Fig. 5G). Surprisingly, however, these enriched transcripts exhibited lower nuclear retention than SG-like structure-depleted transcripts under both control and heat shock conditions (Fig. 5H). Moreover, whereas heat shock significantly increased the nuclear retention of the depleted transcripts, the enriched transcripts remained largely unaffected. Consistent with the canonical role of FgYra1 in mRNA export, *FgYRA1* deletion increased nuclear retention under control conditions; however, this effect was reversed during heat shock (Supplementary Fig. S13A and B). Under heat shock conditions, the loss of *FgYRA1* reduced nuclear retention in both transcript groups; nevertheless, SG-like structure-enriched transcripts consistently displayed lower nuclear retention than SG-like structure-depleted transcripts (Fig. 5I). By comparison, nuclear retention of the total intronic RNA population was further increased by the loss of *FgYRA1* under heat shock conditions (Supplementary Fig. S13C), indicating that transcripts associated with SG-like structures exhibit nuclear retention behaviors distinct from those of the broader transcript population. Taken together, these findings suggest that SG-like structure-associated transcripts represent a unique transcript subset with differential responses to heat shock-induced alterations of FgYra1-dependent RNA export processes.

## Discussion

SGs have garnered attention for their stress-specific response mechanisms, particularly through the tethering of certain transcripts. Nevertheless, their detailed molecular properties remain poorly understood. Here, we report the formation of heat shock-induced FgPab1-positive assemblies and dissect their composition by integrating RNA-seq with proteomic analyses. Under heat shock, we observed a marked accumulation of intronic transcripts within FgPab1-positive structures, potentially linked to the mRNA export factor FgYra1. In addition, total and nuclear RNA-seq analyses reveal widespread changes in transcript nuclear retention during heat shock, supporting an association between these structures and FgYra1-dependent RNA export. Although FgPab1-GFP foci characterized here share several features commonly associated with canonical SGs, including stress-dependent formation, cycloheximide sensitivity, translational repression, and association with multiple SG-associated proteins, the cytoplasmic FgPab1-positive assemblies therefore exhibit the defining characteristics of canonical SGs. However, the molecular identity of the nuclear FgPab1-positive foci remains unresolved. We therefore conservatively retain the term SG-like structures throughout this study when referring to the overall FgPab1-positive assemblies.

Previous studies have established important roles for Pab1 and its interacting proteins, including Lsm7, in SG assembly and stress adaptation [44–47]. These studies have primarily focused on the mechanisms underlying SG formation and their biophysical properties. In the present study, we systematically characterized heat shock-induced SG-like structures through integrated transcriptomic and proteomic approaches. This strategy enabled us to uncover an unexpected association between SG-like structures and the nuclear RNA export machinery, particularly involving FgYra1, and to further investigate the relationship between SG-like structures and transcript nuclear retention during heat shock. Thus, our findings provide novel insights into SG-like structures beyond their assembly mechanisms and highlight a previously unrecognized association with nuclear RNA export dynamics during stress responses.

Although SG formation has been recognized as a universal mechanism across eukaryotes, increasing evidence indicates that SG assembly is highly context-dependent, with different stressors engaging distinct molecular mechanisms to promote granulation [48–50]. Heat shock has been reported to strongly perturb protein homeostasis, translation, and RNA metabolism, all of which have been implicated in SG assembly. Consistent with this notion, we found that heat shock, but not other stresses, induced formation of FgPab1-positive SG-like structures accompanied by substantial translational repression and extensive alterations in nuclear RNA dynamics. Given the established roles of Pab1 in translational regulation and mRNA remodeling [51], these heat shock-associated changes may contribute to a cellular environment that favors the formation or stabilization of FgPab1-positive SG-like structures.

Notably, in other systems—including mammals, plants, and fungi—various stress conditions such as glucose deprivation, osmotic stress, oxidative stress, drought, and heat shock can induce SG formation [8, 9, 52, 53]. Furthermore, *Aspergillus oryzae*, a filamentous fungus closely related to *F. graminearum*, robustly forms SGs in response to diverse stresses. In contrast, under the experimental conditions examined in this study, FgPab1-positive SG-like structures were observed only following heat shock. Although this difference may reflect species-specific aspects of the stress response, alternative explanations, including differences in stress severity, cannot be excluded. In particular, because translational repression was not directly examined under the other stress conditions, we cannot exclude the possibility that these treatments fail to induce detectable SGs because they exert weaker effects on translation under the conditions tested. Unlike other eukaryotes, phytopathogenic fungi are routinely exposed to host-derived stressors such as nutrient limitation, oxidative stress, and osmotic stress, and have evolved complex regulatory systems to tolerate these challenges [18, 54, 55]. However, extreme heat rapidly disrupts essential biological processes [56] and is therefore recognized as a severe stress even by phytopathogens. Thus, further studies will be required to determine the molecular basis for this apparent heat shock specificity and to establish the precise nature of these SG-like structures.

The diverse phenotypic consequences resulting from the loss of individual SG-like structure-associated genes suggest that these factors are unlikely to function as a homogeneous group. Instead, they may participate in distinct functional modules involved in SG-like structure assembly, maintenance, or activity. Defining the molecular organization and hierarchical relationships among these components will require further biochemical and structural characterization of fungal SGs or SG-like structures.

One interesting finding of our study is that the absence of the prion-like domain in FgPab1 did not abolish formation of FgPab1-GFP foci but resulted in a reduced number of GFP foci under heat shock conditions. This observation implies that the prion-like domain is not essential for FgPab1 recruitment to SG-like foci but instead promotes the efficient formation of these structures. Given the established roles of prion-like domains in mediating multivalent interactions and driving biomolecular condensation [6, 57], the FgPab1 prion-like domain may facilitate the efficient formation, stability, or maturation of FgPab1-containing SG-like structures.

A notable feature of the SG-like structure transcriptome is the preferential enrichment of relatively long transcripts. This phenomenon has been consistently reported across eukaryotes, from humans to yeast, as one of the most prominent biophysical characteristics of SG-associated transcripts [12, 58, 59]. It has been proposed that such enrichment is driven by intrinsic RNA properties that promote “RNA self-assembly”, wherein longer transcripts tend to engage in more extensive RNA–RNA interactions, thereby recruiting associated RNA-binding proteins and facilitating granule assembly [59, 60]. In this context, our results align with this model and validate the robustness of the transcriptomic profiles obtained in this study.

Little is known regarding the detailed features of SG-like structure-enriched transcripts. Previous reports have argued that transcript length alone is not a major driver of SG assembly and that *N*^6^-methyladenosine (m^6^A) modification also contributes to transcript targeting to SGs [61, 62]. Current models suggest that SG formation is a downstream consequence of translational repression rather than its direct cause [8, 12]. In contrast, our transcriptomic and GO analyses revealed selective recruitment of a distinct subset of transcripts, particularly those involved in DNA repair, ubiquitination, autophagy, and transcriptional regulation. This suggests that the composition of SG-like structures is shaped not solely by transcript length but also by functional relevance. Consistently, a previous study has demonstrated that only a small subset of mRNAs is specifically targeted to stress-induced RNA granules, implying that bulk translational repression is not the primary determinant of granule formation [58]. Unlike earlier studies that lacked direct comparisons between stressed and unstressed conditions, our parallel analyses enabled identification of SG-like structure-specific transcripts with high confidence, thereby advancing our understanding of the transcriptome associated with SG-like structures.

During heat shock, cells can suppress or remodel mRNA splicing to prioritize the expression of stress-adaptive genes [4, 63]. Consistent with our observations, previous studies have reported that heat shock induces the accumulation of intron-containing transcripts, suggesting that this phenomenon may represent a conserved feature of the cellular stress response [4, 64]. In agreement with these findings, we observed a significant increase in intron abundance under heat shock conditions, including within SG-like structures. Typically, nonspliced mRNAs accumulate in nuclear speckles when pre-mRNA splicing is disrupted [65, 66]. Interestingly, we also observed nucleus-localized FgPab1 foci during heat shock. In this context, we propose a potential link between nuclear RNA export and nucleus-localized FgPab1 foci, further supported by the identification of the nuclear mRNA export adaptor FgYra1 through our AP-MS analysis.

In this study, we demonstrated that *F. graminearum* Yra1 co-localizes with SG-like structures under heat shock conditions. Consistently, ALYREF, the human ortholog of Yra1, has been identified as a component of SGs in response to sodium arsenite treatment [67]. However, upon treatment with tubercidin—an adenosine analog—ALYREF participates in the stress response but does not co-localize with SGs [43]. These observations underscore two noteworthy points. First, different stressors distinctly affect the dynamics and composition of SGs. Second, the mRNA export factor Yra1 likely plays a role in the stress response in a context-specific manner.

Under physiological conditions, Yra1 acts as a nuclear RNA export adaptor by binding mature mRNAs in concert with other components of the TREX complex [16, 68]. Recruitment of Yra1 relies on multiple steps of mRNA maturation, including 5′ capping, polyadenylation, and splicing [69, 70], highlighting that the Yra1-mediated nuclear export system primarily acts on fully processed transcripts. In contrast, upon stress exposure, bulk mRNA export is reduced, facilitating the selective nuclear export of stress-responsive transcripts [16]. Our findings suggest that, under heat shock, SG-like foci exist in the nucleus and sequester FgYra1—or are formed through its recruitment—thereby perturbing the proper function of the nuclear export system. Consistent with this notion, heat shock markedly alters RNA nuclear retention patterns [71] while exerting only a limited effect on overall transcript abundance, indicating that stress predominantly affects RNA trafficking rather than global transcriptional outputs.

Notably, SG-like structure-associated transcripts displayed behaviors distinct from those of the bulk transcriptome. If SG-like structure-associated transcripts simply represented RNAs retained in the nucleus due to impaired function of RNA export factors, they would be expected to exhibit increased nuclear accumulation under heat shock conditions. Importantly, a previous study has shown that RNA export factors (ALYs) positively regulate mRNA nuclear export, where their overexpression enhances export efficiency [72]. However, contradictory observations have also been reported, in which the absence of the export factors causes cytoplasmic mRNA leakage, whereas their overexpression can induce nuclear mRNA retention due to toxicity [73, 74]. These observations suggest that the consequences of RNA export factor perturbation are highly context-dependent.

In our study, transcripts associated with SG-like structures consistently showed lower nuclear retention than SG-like structure-depleted transcripts and remained relatively insensitive to heat shock-induced changes in nuclear retention. Furthermore, although the loss of *FgYRA1* enhanced the nuclear retention of the total intronic RNA population during heat shock, SG-like structure-enriched transcripts retained their distinct distribution patterns. These findings suggest that such transcripts constitute a specialized RNA pool, consistent with our GO analysis, that may be regulated through selective export pathways rather than arising merely as a byproduct of defective RNA export.

A previous study in *S. cerevisiae* has suggested that nuclear RNA export serves as a regulatory checkpoint during heat stress rather than merely a constitutive transport process [71]. Heat stress triggers the dissociation of the export factor from bulk mRNAs, thereby inhibiting general export while allowing selective export and translation of heat shock-responsive transcripts [16]. Additionally, in *Arabidopsis thaliana*, a sterile alpha motif (SAM) domain-containing protein, SAM8, forms nuclear condensates under osmotic stress conditions that selectively sequester RNA export factors, leading to nuclear RNA retention and translational reprogramming [72]. More recently, studies in *S. cerevisiae* have shown that newly transcribed stress-responsive mRNAs can evade SGs and other mRNP condensates, thereby remaining available for preferential translation during stress conditions [75, 76]. Together, these observations support the notion that stress responses involve selective regulation of RNA trafficking and translation, rather than uniform control of all transcripts. In this context, the SG-like structure-associated transcripts identified in our study may represent a distinct transcript subset subject to alternative regulatory mechanisms during heat shock.

In conclusion, our study shows that SG-like structure formation occurs exclusively under heat shock in *F. graminearum*. We provide a comprehensive transcriptomic profile of heat shock-induced SG-like structures and demonstrate a marked enrichment of intron-containing RNAs compared to physiological conditions. Moreover, our results suggest that the physical association of FgYra1 with SG-like foci influences the nuclear RNA export system, thereby contributing to widespread changes in nuclear RNA retention during heat shock. Notably, transcripts associated with SG-like structures exhibit distinct nuclear retention behaviors from those of the bulk transcriptome, supporting the notion that they constitute a specialized RNA pool rather than merely byproducts of defective RNA export. This work experimentally defines the molecular composition of SG-like structures in *F. graminearum*, uncovers their functional consequences, and provides a valuable framework for future exploration of SG or SG-like structure biology in eukaryotes, including filamentous fungi.

## Supplementary Material

gkag840_Supplemental_Files

## Data Availability

Data supporting the findings of this work are available within the paper and its Supplementary Information file. Source data are provided with this paper. RNA-seq data have been deposited in the NCBI Sequence Read Archive under the accession numbers PRJNA1305664 and PRJNA1474590. The mass spectrometry-based proteomics data generated in this study have been deposited to the ProteomeXchange under the accession code PXD080458.
