# Supplementary material for: Interplay between stress granule-like structures and nuclear RNA export in a heat shock-specific manner
Source: Nucleic Acids Res. 2026 Aug 20;54(15):gkag840. doi: 10.1093/nar/gkag840 (PMC13490950; doi:10.1093/nar/gkag840)
Supplement: Supplementary file 1 [file gkag840_supplemental_files.zip › Supplementary data_revised_v2.pdf]

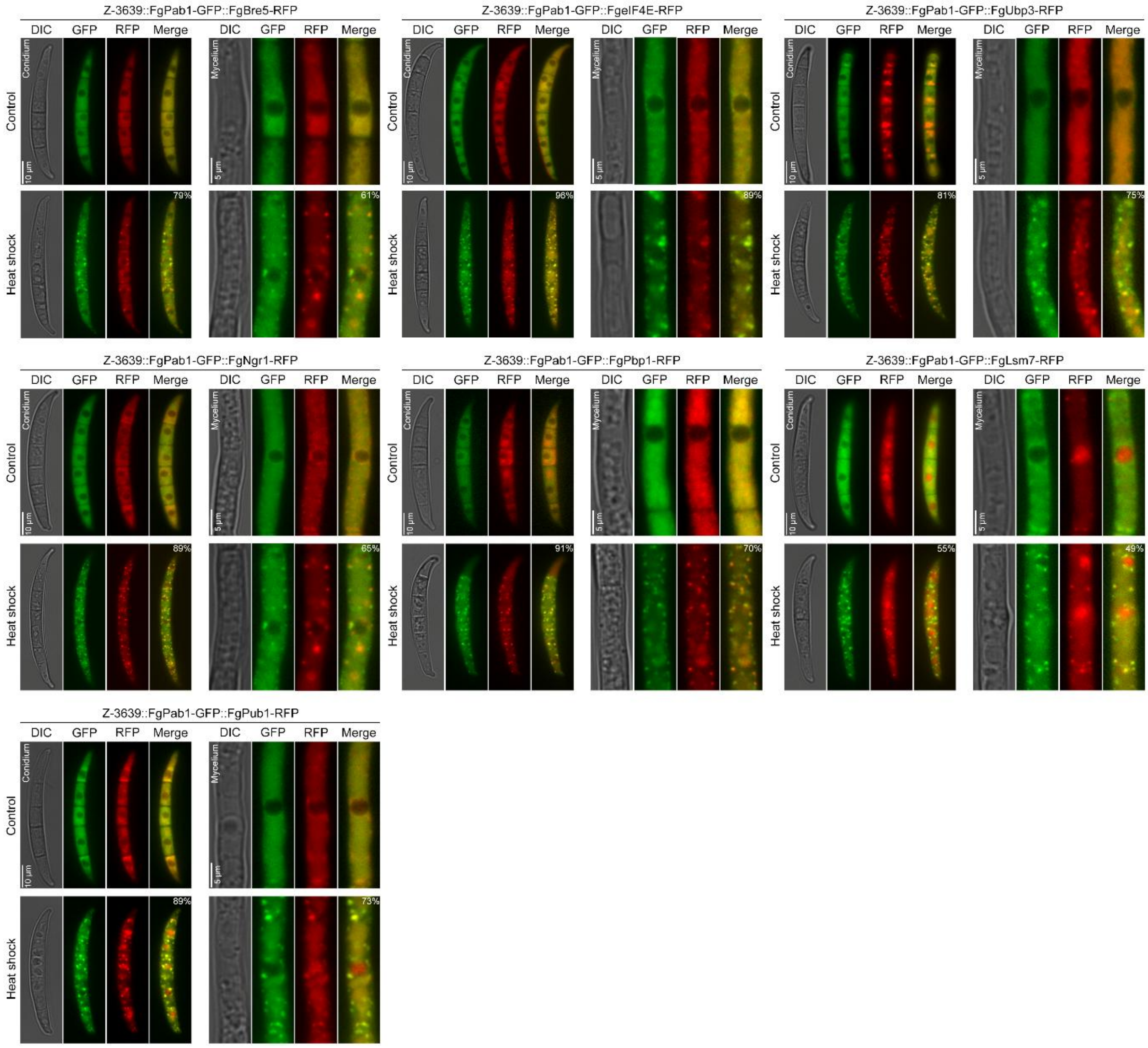

Supplementary Fig. S1: Co-localization analysis of FgPab1-GFP and other SG-associated components tagged with RFP in *F. graminearum*. Representative fluorescence microscopy images showing the subcellular localization of FgPab1-GFP together with the indicated SG-associated proteins (FgBre5, FgeIF4E, FgUbp3, FgNgr1, FgPbp1, FgLsm7, and FgPub1) fused to RFP. Numbers indicate the percentage of FgPab1-GFP foci co-localizing with the corresponding RFP-tagged proteins above background under heat shock conditions.

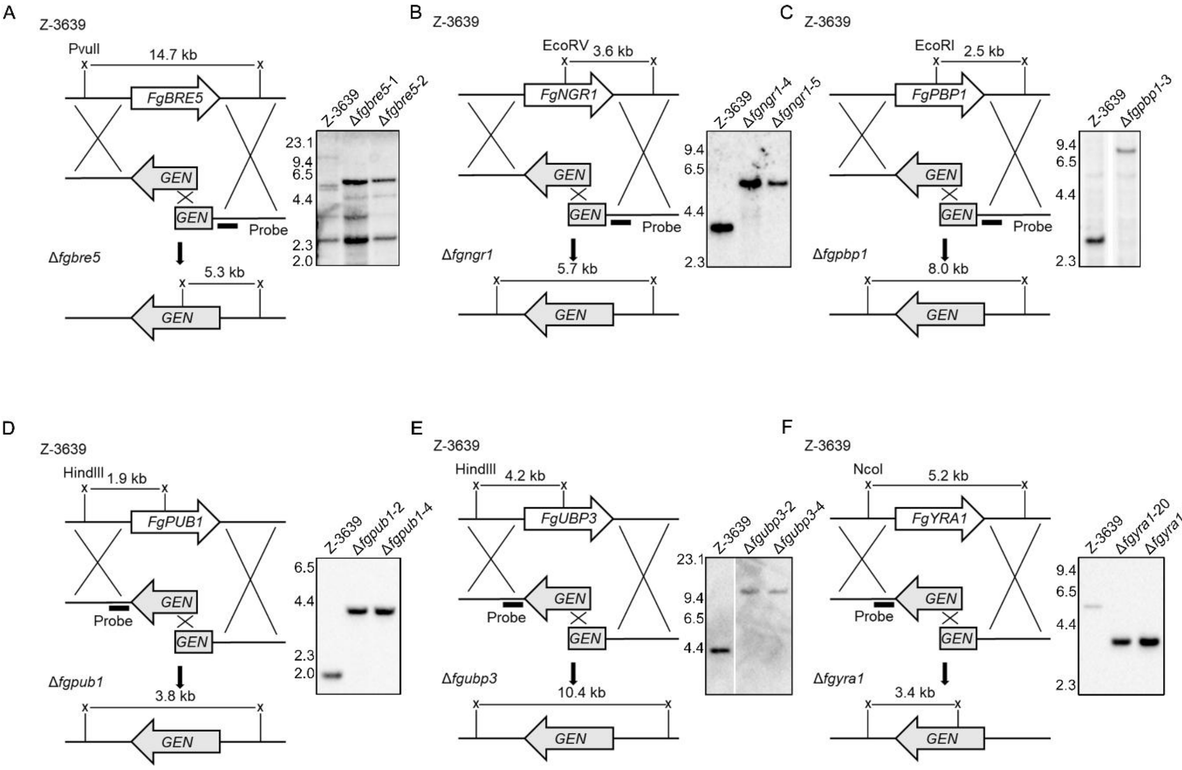

Supplementary Fig. S2: Southern blot hybridization to validate mutant strain construction. (**A**-**F**) The left panel depicts the genetic manipulation strategy, while the right panel shows a representative Southern blot result. *GEN*, geneticin-resistance gene cassette.

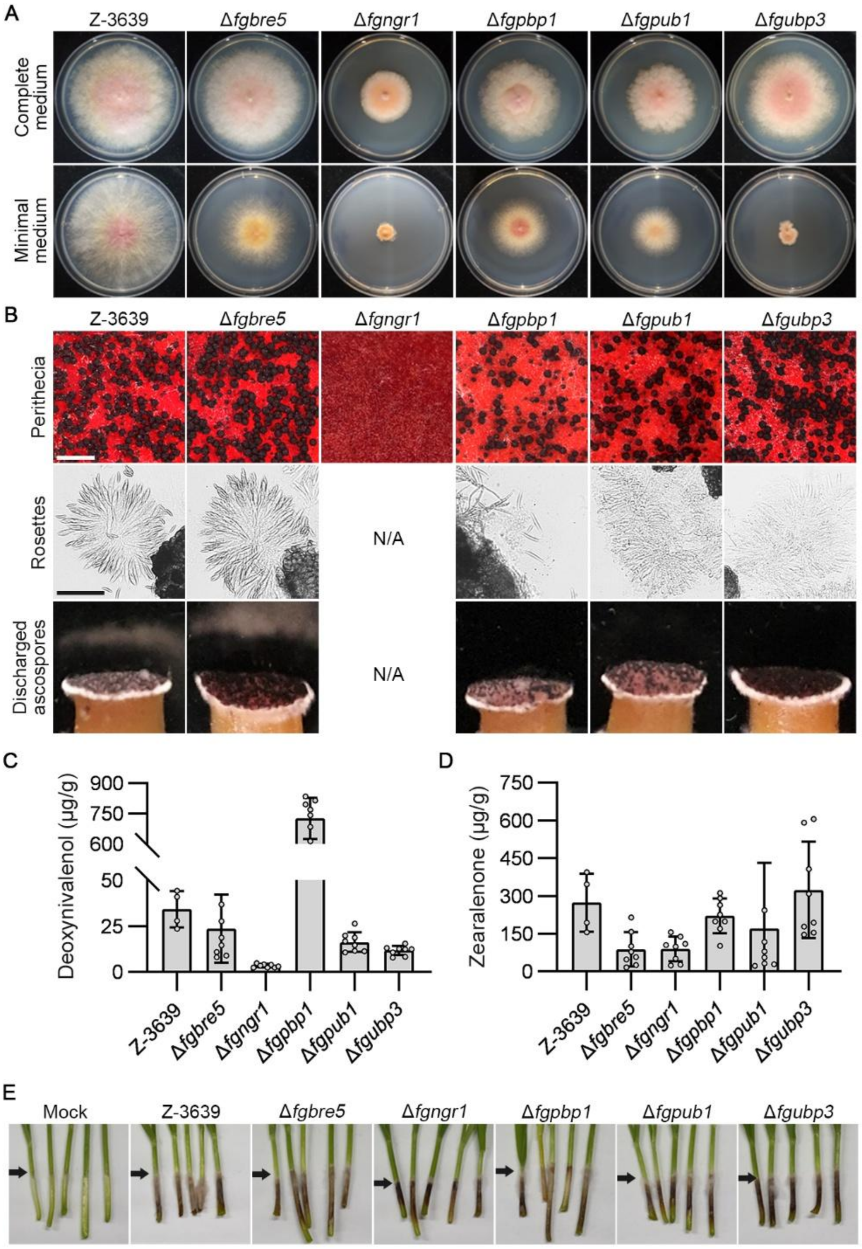

Supplementary Fig. S3: Phenotypic characterization of *F. graminearum* mutant strains. (**A**) Colony morphology of *F. graminearum* mutant strains. Pictures were taken 5 days after inoculation. (**B**) Sexual reproduction on carrot agar medium. Perithecia and rosette structures appeared 7-9 days after sexual induction, and forcibly discharged ascospores were observed after 24 h. Scale bars: black = 1000 μm, white = 100 μm. (**C** and **D**) Mycotoxin production analysis. (**C**) Deoxynivalenol and (**D**) zearalenone were extracted from 3-week-old rice cultures. (**E**) Plant infection assay on wheat coleoptiles. Photographs were taken 7 days after inoculation.

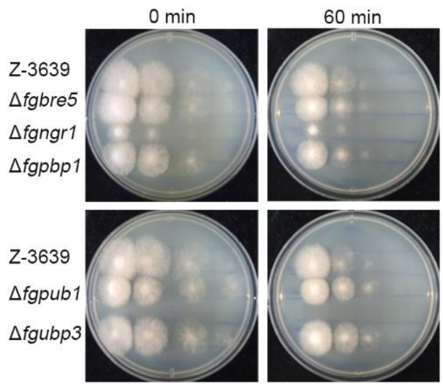

Supplementary Fig. S4: Heat shock sensitivity test. Serial dilutions of conidia were spotted onto complete medium. For heat shock treatment, conidial suspensions were exposed to 42 °C for 60 min.

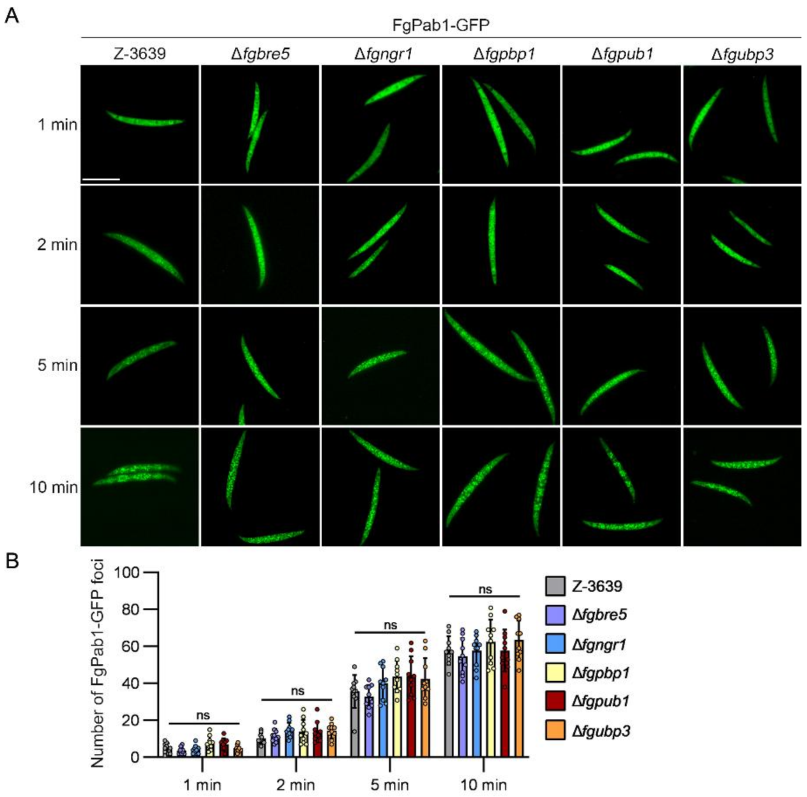

Supplementary Fig. S5: Maintenance of SG-like structures under genetic perturbation. (**A**) Time course analysis of heat shock-induced FgPab1-GFP foci formation in *F. graminearum* mutant strains. Scale bar = 25 µm. (**B**) Quantification of FgPab1-GFP foci per conidium from (**A**).

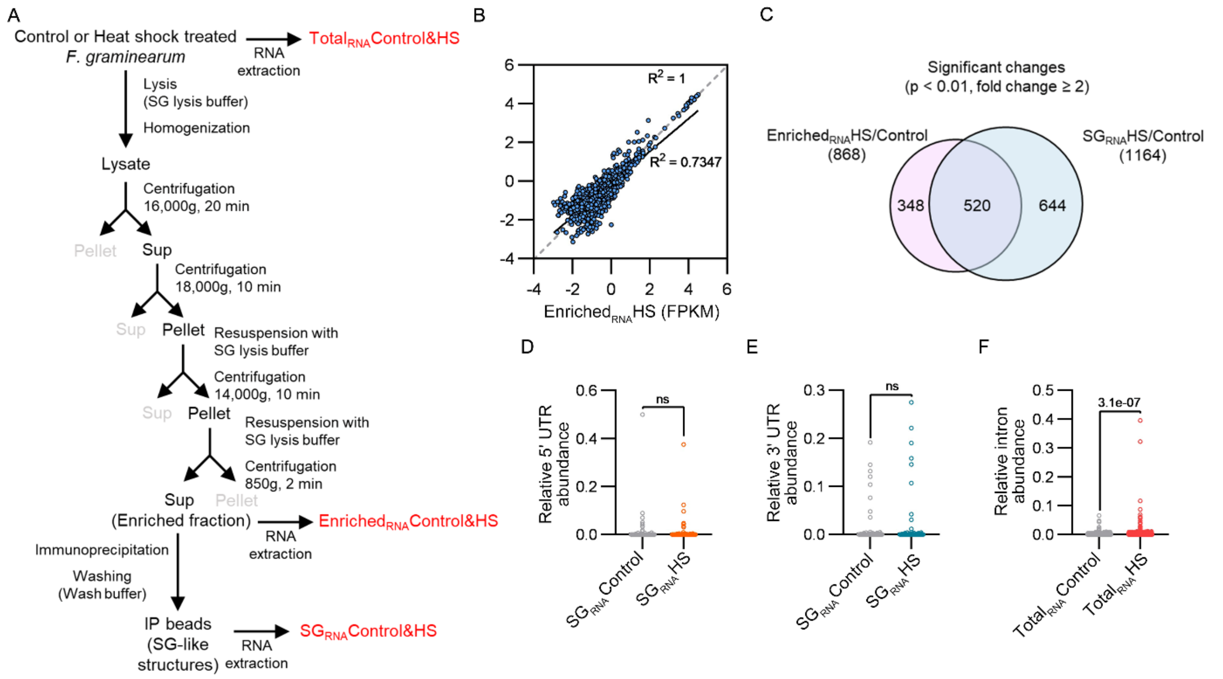

Supplementary Fig. S6: Purification and analysis of *F. graminearum* SG-like structures. (**A**) Schematic of SG-like structure purification. (**B**) Scatter plot depicting transcript abundance (FPKM) between $SG_{RNA}HS$ and $Enriched_{RNA}HS$. (**C**) Overlap of differentially expressed transcripts (DETs) between $Enriched_{RNA}HS$/Control and $SG_{RNA}HS$/Control. (**D**-**F**) Comparisons of (**D**) 5′- or (**E**) 3′-untranslated region (UTR) and (**F**) intron abundance.

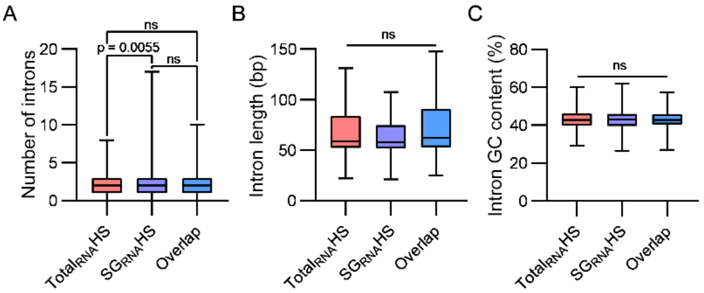

Supplementary Fig. S7: Biophysical features of intronic transcripts enriched within SG-like structures, related to Fig. 3H. (**A**-**C**) Box plots comparing the characteristics of different transcript classes. Shown are (**A**) intron number, (**B**) intron length, and (**C**) intron GC content for each transcript class.

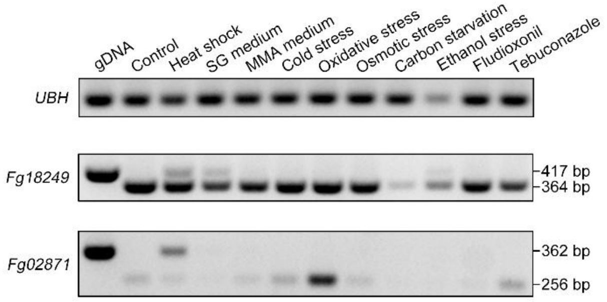

Supplementary Fig. S8: Intron retention analysis under various stress conditions, related to Fig. 4A. Representative RT-PCR analysis showing intron retention in selected transcripts under different stress conditions. Genomic DNA (gDNA) was included as a positive control for intron-containing products. Control indicates the absence of stress treatment. *UBH*, ubiquitin C-terminal hydrolase.

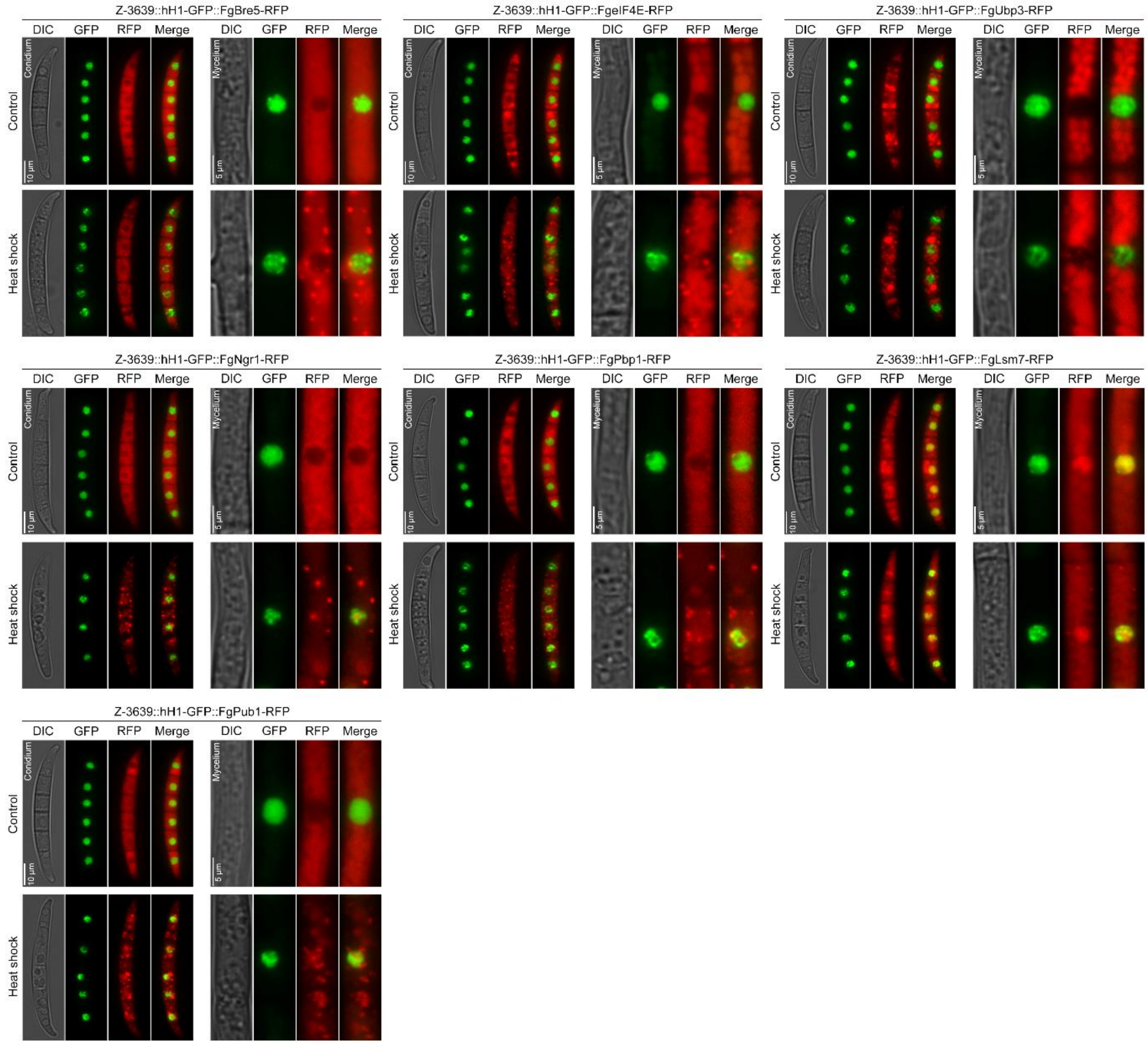

Supplementary Fig. S9: Fluorescence microscopy analysis of histone H1-GFP and SG-associated components tagged with RFP in *F. graminearum*. Representative fluorescence microscopy images showing the localization of histone H1-GFP and the indicated RFP-tagged SG-associated proteins (FgBre5, FgeIF4E, FgUbp3, FgNgr1, FgPbp1, FgLsm7, and FgPub1).

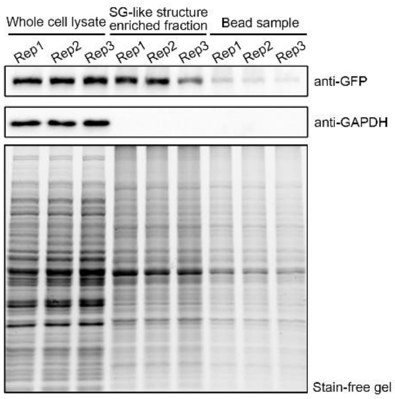

Supplementary Fig. S10: SDS-PAGE and immunoblot analyses of samples used for proteomic analysis. The stain-free gel image shows total protein loaded onto the SDS-PAGE gel. Enrichment of SG-like structures in the SG-like structure-enriched fraction and bead-bound sample was verified by western blotting with anti-GFP and anti-GAPDH antibodies.

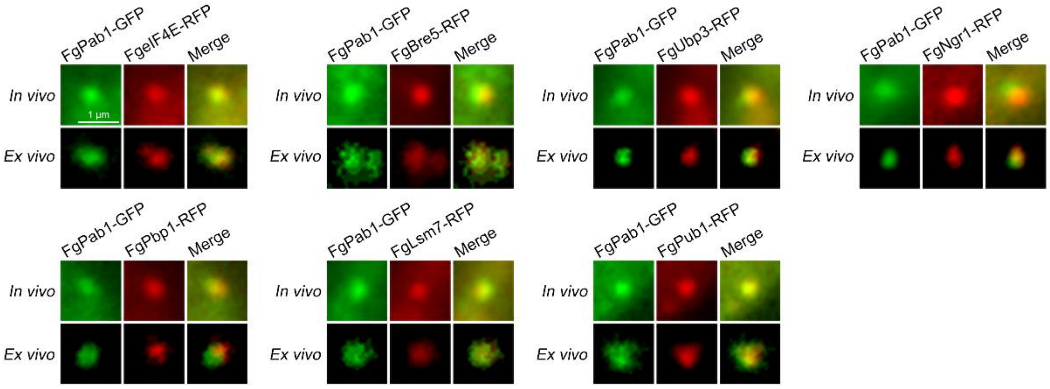

Supplementary Fig. S11: Co-localization analysis of FgPab1-GFP and other SG-associated components tagged with RFP in *F. graminearum* cells (*in vivo*) and cell lysates (*ex vivo*) under heat shock conditions.

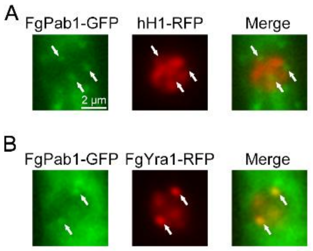

C

Measurement	Percentage
Nuclei containing nuclear FgPab1-GFP foci	47% (47/100)
Nuclear FgPab1-GFP foci co-localized with nuclear speckles	77% (77/100)

Supplementary Fig. S12: Quantification of nuclear FgPab1-GFP foci. (**A** and **B**) Representative fluorescence microscopy images showing nuclear FgPab1-GFP foci in strains expressing (**A**) histone H1-RFP or (**B**) the nuclear speckle marker FgYra1-RFP. (**C**) Quantification from the experiments shown in (**A**) and (**B**).

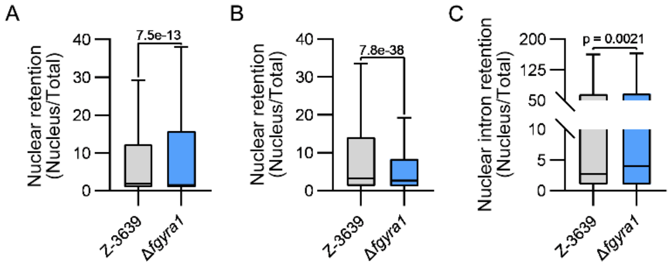

Supplementary Fig. S13: Quantification of nuclear retention rate. (**A** and **B**) Box plots depicting nuclear retention dynamics of RNA populations in the wild-type and Δ*fgyra1* strains under (**A**) control and (**B**) heat shock conditions. (**C**) Box plots showing nuclear retention dynamics of intronic transcripts in the wild-type and Δ*fgyra1* strains under heat shock conditions.

Related to Fig. 4G

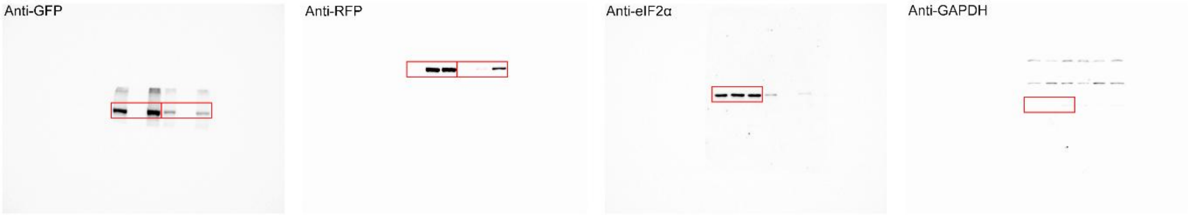

Related to Fig. 5A

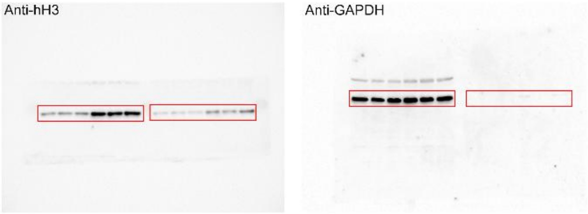

Related to Fig. S10

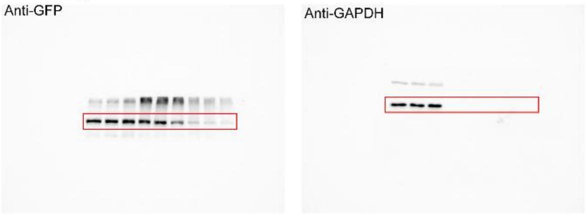

Uncropped scans of all blots

Supplementary Movie S1: Formation of FgPab1-GFP foci during heat shock, related to Fig. 2H. Scale bar = 10 μm
